# *In vivo* efficacy of artemether-lumefantrine and artesunate-amodiaquine for the treatment of uncomplicated falciparum malaria in children: a multisite, open-label, two-cohort, clinical trial in Mozambique

**DOI:** 10.1186/1475-2875-13-309

**Published:** 2014-08-10

**Authors:** Abel Nhama, Quique Bassat, Sónia Enosse, Arsenio Nhacolo, Rosália Mutemba, Eva Carvalho, Eva Naueia, Esperança Sevene, Caterina Guinovart, Marian Warsame, Sergi Sanz, Abdul Mussa, Graça Matsinhe, Pedro Alonso, Armindo Tiago, Eusebio Macete

**Affiliations:** 1 Centro de Investigação em Saúde de Manhiça (CISM), Manhiça, Mozambique; 2 National Institute of Health (INS), Ministry of Health, Maputo, Mozambique; 3 Barcelona Centre for International Health Research (CRESIB, Hospital Clínic-Universitat de Barcelona), Barcelona, Spain; 4 National Malaria Control Programme (NMCP), Ministry of Health, Maputo, Mozambique; 5 World Health Organization (WHO), Maputo, Mozambique; 6 World Health Organization (WHO), Global Malaria Programme, Geneva, Switzerland; 7 Department Salut Pública, Facultat de Medicina, Universitat de Barcelona, Barcelona, Spain; 8 Family Health International (FHI), Maputo, Mozambique

## Abstract

**Background:**

Mozambique adopted artemisinin-based combination therapy (ACT) for the treatment of uncomplicated *Plasmodium falciparum* malaria in the year 2006, and since 2009 artemether-lumefantrine (AL) and artesunate-amodiaquine (ASAQ) have been proposed as alternative first-line treatments. A multicentre study was conducted in five sites across the country to assess the *in vivo* efficacy and tolerability of these two drugs.

**Methods:**

Children aged six to 59 months with uncomplicated malaria were recruited between June 2011 and January 2012 in five sites across Mozambique (Montepuez, Dondo, Tete, Chokwe, and Manhiça), and treated with AL or ASAQ in a non-randomized study. Follow-up was organized following standard WHO recommendations for *in vivo* studies, and included daily visits during the three-day-long supervised treatment course, followed by weekly visits up to day 28. The study primary outcome was the day 28 PCR-corrected early treatment failure (ETF), late clinical failure (LCF), late parasitological failure (LPF), and adequate clinical and parasitological response (ACPR). PCR was performed centrally for all cases of recurrent parasitaemia from day 7 onwards to distinguish recrudescence from re-infection.

**Results:**

Four-hundred and thirty-nine (AL cohort; five sites) and 261 (ASAQ cohort, three sites) children were recruited to the study. Day 28 PCR-corrected efficacy for AL was 96.0% (335/339; 95% CI: 93.4-97.8), while for ASAQ it was 99.6% (232/233; 95% CI: 97.6-99.9). The majority of recurring parasitaemia cases throughout follow-up were shown to be re-infections by PCR. Both drugs were well tolerated, with the most frequent adverse event being vomiting (AL 4.5% [20/439]; ASAQ 9.6% [25/261]) and no significant events deemed related to the study drugs.

**Conclusion:**

This study confirms that both AL and ASAQ remain highly efficacious and well tolerated for the treatment of uncomplicated malaria in Mozambican children. Studies such as these should be replicated regularly in the selected surveillance sentinel sites to continuously monitor the efficacy of these drugs and to rapidly detect any potential signs of declining efficacy to ACT, the mainstay of malaria treatment.

## Background

The last decade has seen a true revolution regarding the diagnosis and treatment of malaria, globally. In sub-Saharan Africa, malaria-endemic countries have progressively replaced, as a consequence of the growing parasite resistance and the associated resurgence in infection rates and malaria-related morbidity and mortality [1], conventional anti-malarial drugs by faster acting and more efficacious anti-malarials. Indeed, artemisinin-based combination therapy (ACT), recommended by the World Health Organization (WHO) since the beginning of this millennium [2], have now been widely adopted in all African countries for the treatment of *Plasmodium falciparum* malaria, a change that has positively contributed to the improved global burden of malaria [3].

In Mozambique, malaria remains a major cause of disease and death, putting an overwhelming pressure on the understaffed and fragile health system [4,5]. National guidelines for the treatment of malaria have experienced various adjustments since the abandoning of chloroquine in the year 2003, and the introduction of sulphadoxine-pyrimethamine (SP) plus amodiaquine. In 2006, this combination was again modified to SP + artesunate and in 2009 to artemether-lumefantrine with artesunate-amodiaquine being considered an alternative first line treatment recommendation [6].

Routine surveillance of *in vivo* efficacy of currently used drugs together with evaluation of the prevalence of molecular markers associated with parasite resistance to anti-malarials is mandatory to assess the adequacy of current treatment recommendations and guarantee a timely response to the emergence of parasite resistance. This is particularly imperative now in relation to the recent documentation in Southeast Asia of the emergence and potential spread of parasite resistance to artemisinins [7-9]. However, only a handful of clinical trials [10-13], and all of them conducted in the same site (Manhiça, southern part of the country), have assessed the in vivo efficacy of ACTs in Mozambique over the last several years, describing efficacy estimates (day 28, PCR-corrected,) for combinations such as artemether-lumefantrine; artesunate-amodiaquine (ASAQ), dihydroartemisinin-piperaquine (DHA-PQP), or SP always exceeding 93% [10,12,13].

In this study, conducted from June 2011 to the end of 2012, in five sentinel sites across the country, the *in vivo* efficacy and safety of artemether-lumefantrine (AL; Coartem™) and ASAQ (Winthrop), the two currently recommended ACT in the country, were assessed for the treatment of uncomplicated *P. falciparum* malaria in Mozambican children between six and 59 months of age.

## Methods

### Study sites and malaria in Mozambique

The study was conducted using the standard WHO *in vivo* efficacy protocol [14] in five hospitals or health centres across Mozambique, namely: 1) *Hospital Rural de Montepuez*, in Cabo Delgado province (northern region); 2) *Centro de Saúde de Dondo*, in Sofala province (central region); 3) *Hospital provincial de Tete*, in Tete province (central region); 4) *Hospital rural de Chokwe*, in Gaza province (southern region); and 5) *Hospital distrital de Manhiça*, in Maputo province (southern region). Malaria transmission in Mozambique is perennial, with a peak transmission period normally coinciding with the rainy season, from November to April. Study cohort 1 testing AL began in June 2011 and involved the five sites, while study cohort 2 assessing the combination ASAQ started in August 2012, once the follow-up for the first cohort had been concluded, and was only conducted in three of the five sites (Montepuez, Dondo and Chokwe).

### Patients

The study population comprised children aged six to 59 months with microscopically confirmed, acute uncomplicated malaria. Other inclusion criteria included body weight ≥5 kg, the presence of fever (≥37.5°C axillary) or a history of fever in the preceding 24 hours, *P. falciparum* malaria mono-infection with an asexual blood density ≥2,000/μL and <200,000/μL, and the absence of severe signs of complicated malaria as defined by WHO [15]. Key exclusion criteria included mixed malarial infections, haemoglobin <5 g/dL, severe malnutrition, intake of anti-malarials within the preceding seven days, ongoing prophylaxis in HIV-positive patients with cotrimoxazole or the intake of any other drug with anti-malarial activity, and any serious underlying disease. Patients satisfying the inclusion criteria were enrolled if the parent/guardian signed a detailed written informed consent.

### Treatment

Eligible patients were consecutively assigned to the cohort and treated with AL (cohort 1) or ASAQ (cohort 2). AL (Coartem™, Novartis, each tablet contains 20 mg artemether and 120 mg lumefantrine) was administered twice daily for three days (six doses in total) with dosage determined according to body weight: one tablet for children 5 to <15 kg, two tablets for children 15 to <25 kg, and three tablets children 25 to <35 kg. ASAQ (Winthrop™, Sanofi Aventis, each tablet contains 25 mg artesunate and 67.5 mg amodiaquine) was administered once daily according to body weight: one tablet tablet for children <9 kg, two tablets for children 9–17.9 kg; and four tablets for children >18-35 kg. All treatments were directly observed for a minimum of 30 min. Vomiting occurring within the first 30 min implied the repetition of the full dose of treatment. For those patients living far away from the health facilities, and for which direct observation of the evening doses of AL was challenging, admission was offered for the first three days of the study.

Antipyretics, such as paracetamol, were used to control fever > = 38°C. In the event of severe malaria or danger signs, the patient was hospitalized and received intravenous quinine, according to the national malaria treatment policy. Rescue therapy according to national malaria treatment guidelines was also administered in cases of early or late treatment failure with parenteral quinine [16].

### Evaluation

Follow-up visits took place on days 1, 2, 3, 7, 14, 21 and 28 after enrolment or at any time point whenever the child was sick. Patients who prematurely discontinued either study drug or the study were excluded from the study. Vital signs and body temperature were assessed during each follow-up visit. Adverse events were recorded and assessed for severity and association with study medication.

Thick and thin Giemsa-stained blood slides were prepared before each dose was administered and at every follow-up visit of days 2, 3, 7, 14, 21, and 28. Slides were examined by two independent microscopists and considered negative if no parasites were seen after examination of 200 oil-immersion fields in a thick blood film. Parasite density was estimated by counting the number of asexual parasites in 200 white blood cells (WBC), assuming a standard WBC count of 8,000 /μl. Species determination (and thus conformation of mono-infection) was made based on assessment of thin films. Blood spots for PCR analysis were collected from every patient using 3 M Whatman™ filter papers at baseline and at days 7, 14, 21 and 28, day of treatment failure or at any other unscheduled visit, and subsequently stored in plastic zip bags containing silica gel dessicant. PCR was performed centrally for all cases of recurrent parasitaemia from day 7 onwards, including DNA extraction using a QIAamp DNA Mini Kit (Qiagen), and investigation of the three polymorphic genetic markers MSP1, MSP2, and GluRP, which were used to distinguish recrudescence from new infections, according to WHO recommended procedures [17]. Recrudescence was defined as at least one identical allele for each of the three markers in the pre-treatment and post-treatment samples. New infections were diagnosed when all alleles for at least one of the markers differed between the two samples. Cases with new infection were excluded from the analysis.

### Study outcomes

The primary efficacy outcomes were the PCR-corrected early treatment failure (ETF), late clinical failure (LCF), late parasitological failure (LPF) and adequate clinical and parasitological response (ACPR) at day 28. Secondary outcomes included 28 day-uncorrected ACPR (crude efficacy), safety and tolerability profiles, time to parasite, fever and gametocyte clearance, and haemoglobin changes from baseline to day 28.

### Data management and statistical analysis

Data were recorded using specifically designed, standardized case report forms based on those proposed by WHO [14]. All study questionnaires were doubled-entered into a study-specific database created using open clinica software (OpenClinica Enterprise - Electronic Data Capture Software for Clinical Trials version 3.1.2, OpenClinica LLC, Waltham, MA, USA). Two populations were defined for the analysis: the intent-to-treat (ITT) population (safety population) comprised all patients who received ≥ one dose of study medication and underwent at least one post-baseline safety assessment. Efficacy was calculated in the according-to-protocol population (ATP), which included all patients fulfilling the protocol eligibility criteria, having completed the three-day course of study medication, accomplishing the day-28 assessment and having an evaluable PCR in case of recurrent parasitaemia. Cure rates were calculated as the number of patients with clinical and parasitological cure by day 28 divided by the total number of patients who could be evaluated. Additionally, Kaplan-Meier estimates of the cumulative risk of failure were computed for day 28. For such an analysis, losses to follow-up and study withdrawals were censored on the last day of follow-up. Cases with re-infections were also censored from the analysis. Statistical analyses were done with Stata 13 (Stata Corp, College Station, TX, USA), and the statistical significance level was set at 5%. No formal comparisons were made between the two study cohorts.

### Sample size calculations

The only available published data regarding the efficacy of the two studied ACT in Mozambique come from previous studies conducted in Manhiça, where AL was shown to have an efficacy ranging from 96.9% [13] to over 98% [12] and ASAQ of 97.2% [10]. Sample size calculations were based on WHO-proposed methodologies [14] using a slightly more conservative expected efficacy estimate (95%) and a level of precision around the estimate of 5%. To achieve this, and with an expected loss to follow-up rate of 20% on day 28, a minimum 87 patients would need to be recruited at each of the sites and for each of the two study arms.

### Ethical considerations

The protocol was approved by the National Mozambican Ethics Review Committee (Ref 134/CNBS/11) and the Hospital Clínic of Barcelona Ethics Review Committee. The trial was conducted according to Good Clinical Practice guidelines. Written consent was obtained from the parents/guardians of the study children. The clinical-trial identifier is NCT02168569 (http://www.clinicaltrials.gov).

## Results

### Trial profile and baseline characteristics

**Figure 1:**
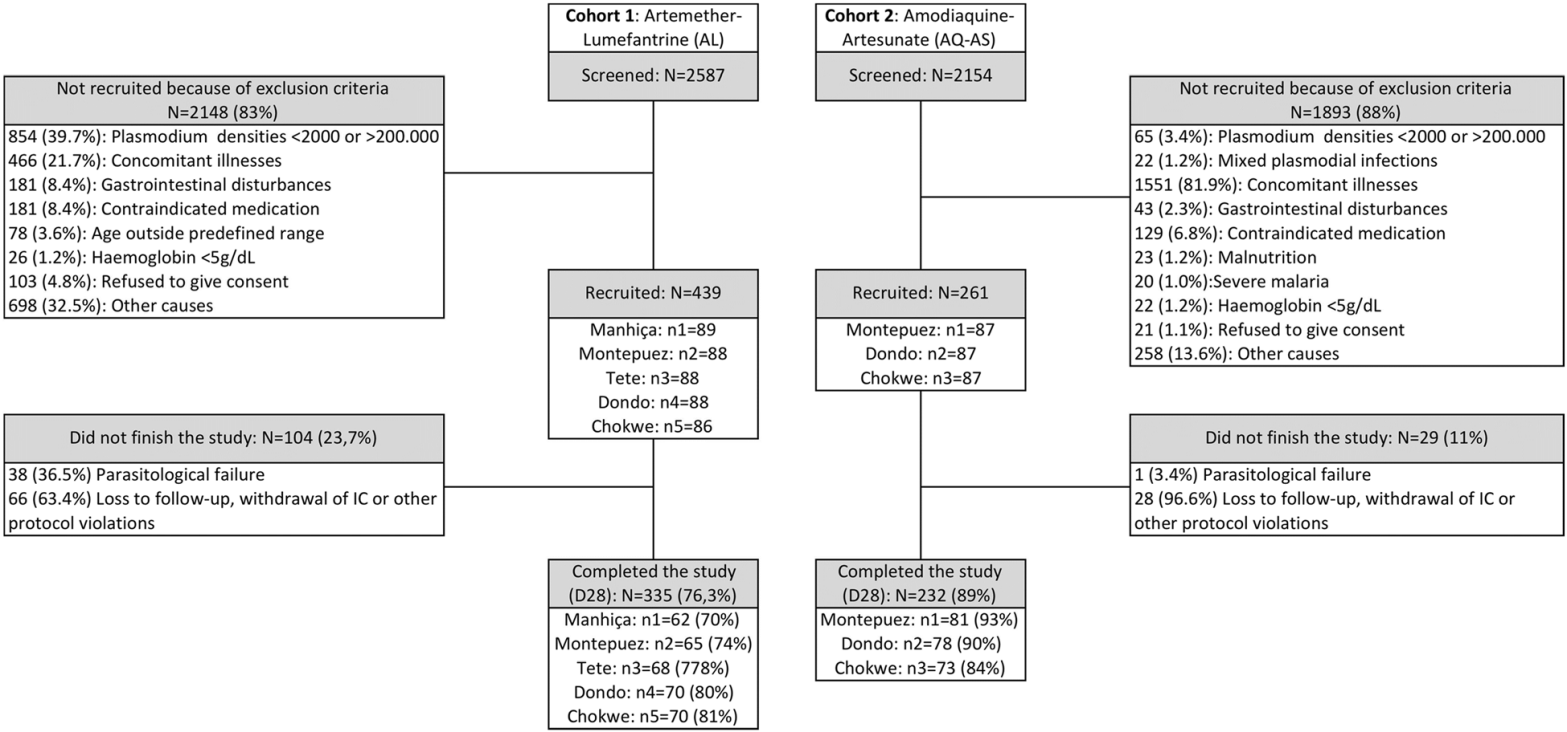
Study profile in both study cohorts.

Between June 2011 and January 2012, both cohort 1 (AL) and cohort 2 (ASAQ) were recruited and followed up to 28 days. Some 2,587 febrile children were screened for cohort 1, of which 439 (16.9%) ended up being recruited and 335 (76.3%) completed the study, with or without a recurring parasitaemia. For cohort 2, 2,154 febrile children were screened, 261 (12.1%) recruited and 232 (89.0%) successfully completed the study, with or without a recurring parasitaemia (Figure 1). Main reasons for exclusion in both cohorts was the absence of malaria parasites at screening and/or the presence of concomitant illnesses. The ITT and ATP populations for cohort 1 included 439 and 335 individuals, respectively, whereas for cohort 2 these numbers were 261 and 232, respectively. Table 1 summarizes the baseline characteristics for both cohorts, which were comparable in terms of gender, mean age, weight, and malarial infection.

**Table 1:** **Baseline characteristics of enrolled subjects according to study cohort** (**treatments received AL and ASAQ**) **at various sites in Mozambique**

	**Study cohort 1 (AL)**	**Study cohort 2 (AS-AQ)**
**Variable**	**N** = **439**	**N** = **261**
Female gender n (%)	197 (45)	137 (52)
Age in years (mean ± SD)	2.5 ± 1.2	2.5 ± 1.3
Weight in kg (mean ± SD)	11.8 ± 2.9	12.2 ± 3.7
Fever n (%)	319 (73)	201 (77)
Temperature in°C (mean ± SD)	38.0 ± 1.0	38.1 ± 0.9
Parasite density geometric mean (range)	30,094 (2,178-19,0011)	34,055 (2,417-213,394)
Hb in g/dL (mean ± SD)	9.2 ± 2.0	9.0 ± 1.8

### Efficacy

**Figure 2:**
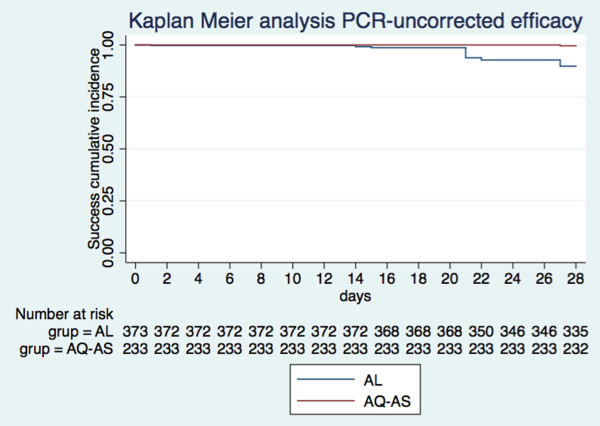
**Kaplan Meier curves showing the treatment success cumulative proportion for each treatment cohort until day 28 (PCR uncorrected) in the ATP population.**

**Figure 3:**
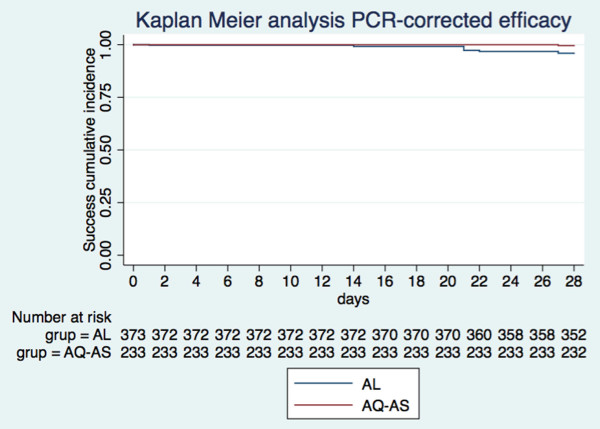
**Kaplan Meier curves showing the treatment success cumulative proportion for each treatment cohort until day 28 (PCR-corrected) in the ATP population.**

During the 28-day follow-up period, 66 patients (cohort 1; 15%) and 28 patients (cohort 2; 10.7%) did not successfully complete the study on account of loss to follow-up, withdrawal of consent or other protocol violations (Figure 1). Table 2 and Figures 2 and 3 summarize both cohort 1 and cohort 2 treatment outcomes. The day-28 PCR-uncorrected cure rate (i.e., the proportion of patients with ACPR, ATP population) was 89.6 (335/374; 95% CI 86.0-92.5) for AL, and 99.6 (232/233; 95% CI 97.6-99.9) for ASAQ. Of the 38 cases of recurring parasitaemia in cohort 1, 25 (65.8%) proved to be new infections according to PCR, yielding a day-28 PCR-corrected cure rate of 96.0 (335/349; 95% CI 93.4-97.8). In the ASAQ, only one recurring parasitaemia was detected, which proved to be a recrudescence of the original infection according to PCR. Thus, day-28 PCR-corrected cure rate was identical to the uncorrected one (99.6 [232/233; 95% CI 97.6-99.9]). All patients in both cohorts cleared their parasitaemia by day 3 following treatment, 100% patients treated with ASAQ by the end of the first 24 hours of follow-up, while 5.0% (22/439) of the patients treated with AL took longer than 24 hours to clear parasitaemia (of these, 77.2% [17/22] cleared parasitaemia on day 2, while 22.7% [5/22] on day 3).

**Table 2:** **Treatment outcomes on day 28**, **according to study cohort** (**AL or ASAQ**) **at various sites in Mozambique**

	**Study site**					
**Cohort 1**: Artemether-Lumefantrine	**Montepuez**	**Dondo**	**Chokwe**	**Manhiça**	**Tete**	**TOTAL**
**Variable**	**N** = **88**	**N** = **88**	**N** = **86**	**N** = **89**	**N** = **88**	**N** = **439**
ACPR^a^ (uncorrected) n	65	70	70	62	68	335
ETF^b^ n	0	0	1	0	0	1
LCF^c^ n	4	4	0	0	0	8
LPF^d^ n	9	6	6	5	4	30
New infections (with PCR) n	9	7	4	3	2	25
Recrudescences (with PCR) n	4	3	2	2	2	13
No treatment outcome (loss to follow-up or withdrawn) n	10	8	9	22	16	66
PP^e^ day-28 efficacy (PCR-uncorrected) n/N (95%CI)	65/78 (83.3) [73.2-90.8]	70/80 (87.5) [78.2-93.8]	70/77 (90.9) [82.2-96.3]	62/67 (92.5) [83.4-97.5]	68/72 (94.4) [86.4-98.5]	335/374 (89.6) [86.0-92.5]
PP day-28 efficacy (PCR-corrected) n/N (95%CI)	65/69 (94.2) [85.8-98.4]	70/73 (95.9) [88.5-99.1]	70/73 (95.9) [88.5-99.1]	62/64 (96.9) [89.2-99.6]	68/70 (97.) [90.1-99.7]	335/349 (96.0) [93.4-97.8]
**Cohort 2**: Artesunate-amodiaquine	**Montepuez**	**Dondo**	**Chokwe**	**Manhiça**	**Tete**	**TOTAL**
**Variable**	**N** = **87**	**N** = **87**	**N** = **87**	**N** = **0**	**N** = **0**	**N** = **261**
ACPR^a^ (uncorrected) n	81	78	73	NA	NA	232
ETF^b^ n	0	0	0	NA	NA	0
LCF^c^ n	0	0	0	NA	NA	0
LPF^d^ n	0	0	1	NA	NA	1
New infections (with PCR) n	0	0	0	NA	NA	0
Recrudescences (with PCR) n	0	0	1	NA	NA	1
No treatment outcome (loss to follow up or withdrawn) n	6	9	13	NA	NA	28
PP day-28 efficacy (PCR-uncorrected) n/N (95%CI)	81/81 (100) [NA]	78/78 (100) [NA]	73/74 (98.6) [92.7-99.9]	NA	NA	232/233 (99.6) [97.6-99.9]
PP day-28 efficacy (PCR-corrected) n/N (95%CI)	81/81 (100) [NA]	78/78 (100) [NA]	73/74 (98.6) [92.7-99.9]	NA	NA	232/233 (99.6) [97.6-99.9]

^a^ACPR:adequate clinical and parasitological response; ^b^ETF: early treatment failure; ^c^LCF: late clinical failure; ^d^LPF: late parasitological failure; ^e^PP: per protocol; NA: not applicable.

### Tolerability and safety

**Figure 4:**
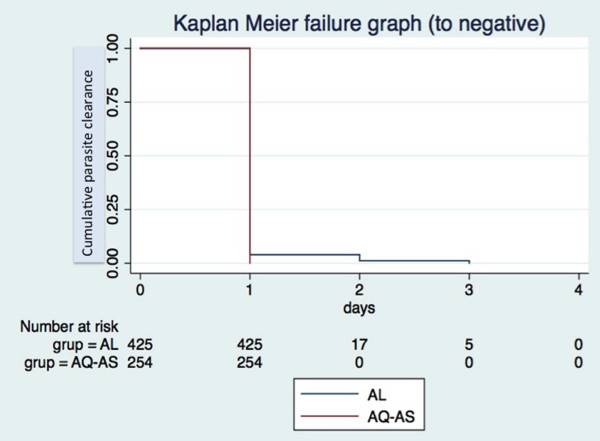
Kaplan Meier curve showing time to negative parasitaemia according to study cohorte.

**Figure 5:**
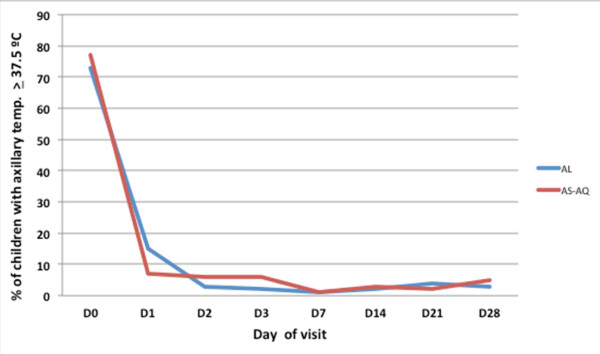
**Proportion of children with fever (axillary temperature ≥37.5°C) according to day of visit and treatment cohort.**

Fever, present at recruitment in about three-quarters of all study patients, receded rapidly during the first 72 hours of follow-up, with all patients being afebrile by day 7 of follow-up (Figures 4 and 5). Tolerability of the two drugs, as judged by solicited adverse events during drug intake, was good (Table 3). Some patients reported vomiting (20/439, 4.5% in the AL cohort; 25/261, 9.6% in the ASAQ group) during the three days of treatment, and the occurrence of other solicited adverse events was rare. All the vomiting episodes (with the exception of two episodes in different children, both occurring in the ASAQ group during the first day of treatment and after >1 hour post treatment, deemed unrelated to the study drug) occurred within the first 30 minutes of treatment, and thus required re-dosing. No patient had repeated vomiting episodes, and no rescue treatment was required on account of vomiting. Six urticarial-like episodes (2/6 [33.3%] in the AL group, 4/6 [66.6%] in the ASAQ group) occurred in both groups, being all of them self-limited, transient and of mild nature. In the majority of these cases (5/6; 83.3%) these events were judged unrelated to the study drug, as alternative explanations (“atopy”; “eczema”; “scabies-related rash” (twice); “viral rash”) were provided, although in one case (16.7%; post AQAS), investigators deemed it “possibly” drug-related, and the skin manifestations disappeared rapidly without requiring any treatment.

**Table 3:** **Cumulative adverse events related to tolerability during the three days of treatment for both study cohorts** (**AL or ASAQ**) **at various sites in Mozambique**

	**Study site**					
**Cohort 1**: Artemether-lumefantrine	**Montepuez**	**Dondo**	**Chokwe**	**Manhiça**	**Tete**	**TOTAL**
**Variable**	**N** = **88**	**N** = **88**	**N** = **86**	**N** = **89**	**N** = **88**	**N** = **439**
Vomiting post dosing 1,2 or 3 n (%)	1 (1.1)	4 (3.2)	1 (1.1)	10 (11.2)	4 (3.2)	20 (4.5)
Diarrhoea n (%)	0 (0)	0 (0)	0 (0)	0 (0)	1 (1.1)	1 (0.2)
Weakness n (%)	0 (0)	0 (0)	0 (0)	0 (0)	1 (1.1)	1 (0.2)
Pruritus n (%)	0 (0)	0 (0)	0 (0)	0 (0)	0 (0)	0 (0)
Urticaria n (%)	1 (1.1)	0 (0)	0 (0)	1 (1.1)	0 (0)	2 (0.5)*
**Cohort 2**: Artesunate-amodiaquine	**Montepuez**	**Dondo**	**Chokwe**	**Manhiça**	**Tete**	**TOTAL**
**Variable**	**N** = **87**	**N** = **87**	**N** = **87**	**N** = **0**	**N** = **0**	**N** = **261**
Vomiting post dosing 1,2 or 3 n (%)	11 (12.6)	3 (3.5)	11 (12.6)	NA	NA	25 (9.6)
Diarrhoea n (%)	0 (0)	0 (0)	6 (6.9)	NA	NA	6 (2.3)
Weakness n (%)	0 (0)	0 (0)	3 (3.5)	NA	NA	3 (1.1)
Pruritus n (%)	0 (0)	0 (0)	2 (2.3)	NA	NA	2 (0.8)
Urticaria n (%)	0 (0)	0 (0)	4 (4.6)	NA	NA	4 (0.9)**

*Urticaria in AL group deemed NOT related with study medication in both cases.

**Urticaria in AS-AQ deemed related to study medication in one of these 4 cases.

*NA*: Not applicable.

Haemoglobin recovery from day 0 to day 28 occurred slowly in both groups (mean increase in the AL cohort being 1.0 (SD 4.2) and in the ASAQ cohort being 1.6 (SD 1.7) (Table 4). Seven serious adverse events (SAE) were documented in the study: four in the AL group (three severe malaria cases and a Kwashiorkor with pellagra diagnosis) and three in the ASAQ group (two severe malaria cases one of which with concomitant severe pneumonia), none deemed by the investigators related to the study drugs. Six of the seven SAEs recovered completely, but an 11-month-old female child in the Montepuez site died after a severe malaria episode, which resolved adequately at hospital. The death occurred at home, on the third day of follow-up, and according to the verbal autopsy subsequently performed, appeared related to the uncontrolled administration of traditional medicine.

**Table 4:** **Change in haemoglobin** (**g**/**dL**) (**difference day 0 *vs *day 28**) **according to study cohort** (**AL or ASAQ**) **at various sites in Mozambique**

		**Study site**					
	**Variable**	**Montepuez**	**Dondo**	**Chokwe**	**Manhiça**	**Tete**	**TOTAL**
**Cohort 1** (artemether-lumefantrine)	Change in haemoglobin g/dL (diff. day 28 vs. day 0) arithmetic mean (SD) [n]	1.2 (1.8) [71]	1.7 (2.0) [75]	1.3 (2.2) [72]	0.5 (1.9) [73]	0.3 (8.6) [69]	1.0 (4.2) [360]
**Cohort 2** (artesunate-amodiaquine)	Change in Haemoglobin g/dL (diff. D28 vs. D0) Arithmetic mean (SD) [n]	1.9 (1.5) [83]	1.8 (1.8) [78]	0.9 (1.6) [76]	N/A	N/A	1.6 (1.7) [237]

## Discussion

This study describes the 28-day in vivo efficacy of AL and ASAQ across several sentinel sites in Mozambique. These five sites were thought to be a good geographical representation of the variability of malaria endemicity throughout the country. All sites followed the standard, WHO-recommended, *in vivo* efficacy protocol [14], including the use of molecular techniques to differentiate a recurring parasitaemia and a recrudescence or a new infection [17]. Although it was not possible to test the second combination (ASAQ) in all five sites, the results presented confirm that both drugs remain efficacious and well-tolerated regimens in Mozambique. In this study, both drugs appeared to be safe and well-tolerated, perhaps with the exception of a non-negligible incidence of vomiting associated with the use of ASAQ, something that has previously been reported in relation to the use of amodiaquine on its own [18] or in combination with artesunate [19,20]. Haemoglobin recovery from day 0 to day 28 occurred swiftly in both cohorts and in a comparable manner to what other authors have described when using these two drugs [10], and the occurrence of other AEs or SAEs was rare. These reassuring data add to the well-documented, good safety profiles of ACT.

In terms of efficacy, PCR-corrected cure rates for AL (96.0%) and ASAQ (99.6%) remain high and adequate according to WHO recommendations. As this study was not designed as a direct comparison between the two cohorts, caution needs to be taken in terms of comparing both drugs’ efficacy estimates. This relatively lower efficacy of AL, in comparison to ASAQ and also to previous historical estimates [10,12,13], could be a first signal of a potential decline of AL efficacy in Mozambique. However, the confirmation that all patients in the AL group had cleared parasitaemia by day 3 (72 hr post treatment), a proxy harbinger of artemisinin resistance as proposed by some authors [21,22], is reassuring. However, other factors such as the challenges in directly observing the evening treatment in the group receiving AL, may have also contributed to these differences. Continuous monitoring throughout the country of AL *in vivo* efficacy is thus necessary to allow an early detection of further signs of AL declining efficacy.

## Conclusion

This multisite efficacy study conducted in five sites across Mozambique confirmed that AL and ASAQ are still highly efficacious and well tolerated. Studies such as this one should be replicated regularly nationwide to continuously monitor the efficacy of these drugs and to rapidly detect any potential signs of declining efficacy to ACT, the mainstay of malaria treatment.

## Competing interests

The authors have declared they have no competing interests.

## Authors’ contributions

All authors contributed to the design of the study and assisted with data interpretation. AN, QB, SE, RM, EC, ES, SS, EN; CG, AM, AT, and EM coordinated the study and supervised the enrolment and follow-up of patients. SS and ArN provided biostatistical support. PA and MW provided overall guidance. All authors participated in the preparation of the manuscript and approved the final version.
